# Causal associations of tea intake with COVID-19 infection and severity

**DOI:** 10.3389/fnut.2022.1005466

**Published:** 2023-01-04

**Authors:** Ancha Baranova, Yuqing Song, Hongbao Cao, Weihua Yue, Fuquan Zhang

**Affiliations:** ^1^School of Systems Biology, George Mason University, Manassas, VA, United States; ^2^Research Centre for Medical Genetics, Moscow, Russia; ^3^Peking University Sixth Hospital/Institute of Mental Health, Beijing, China; ^4^NHC Key Laboratory of Mental Health (Peking University), National Clinical Research Center for Mental Disorders (Peking University Sixth Hospital), Beijing, China; ^5^PKU-IDG/McGovern Institute for Brain Research, Peking University, Beijing, China; ^6^Chinese Institute for Brain Research, Beijing, China; ^7^Institute of Neuropsychiatry, The Affiliated Brain Hospital of Nanjing Medical University, Nanjing, China; ^8^Department of Psychiatry, The Affiliated Brain Hospital of Nanjing Medical University, Nanjing, China

**Keywords:** COVID-19, tea intake, Mendelian randomization, genetics, *Camellia sinensis*

## Abstract

Tea ingredients can effectively inhibit SARS-CoV-2 infection at adequate concentrations. It is not known whether tea intake could impact the susceptibility to COVID-19 or its severity. We aimed to evaluate the causal effects of tea intake on COVID-19 outcomes. We performed Mendelian randomization (MR) analyses to assess the causal associations between tea intake (*N* = 441,279) and three COVID-19 outcomes, including SARS-CoV-2 infection (122,616 cases and 2,475,240 controls), hospitalized COVID-19 (32,519 cases and 2,062,805 controls), and critical COVID-19 (13,769 cases and 1,072,442 controls). The MR analyses indicated that genetic propensity for tea consumption conferred a negative causal effect on the risk of SARS-CoV-2 infection (OR: 0.87, 95% confidence interval (CI): 0.78–0.97, *P* = 0.015). No causal effects on hospitalized COVID-19 (0.84, 0.64–1.10, *P* = 0.201) or critical COVID-19 (0.73, 0.51–1.03, *P* = 0.074) were detected. Our study revealed that tea intake could decrease the risk of SARS-CoV-2 infection, highlighting the potential preventive effect of tea consumption on COVID-19 transmission.

## Introduction

COVID-19 has caused millions of deaths and is an ongoing threat to global public health. A collection of risk and protective factors have been identified to be associated with COVID-19 (1–10). Meanwhile, COVID-19 can give rise to a myriad of post-COVID-19 consequences (11–15). The continued effectiveness of the current generation of COVID-19 vaccines and the possible acquisition of resistance to antivirals remain a concern. Natural products, especially those present in the staple diet, may have the potential to provide a cost-efficient avenue to decrease the risk of COVID-19 transmission or symptomatic relief (16).

In ancient China, local outbreaks of infectious disease were common; hence, herbal or other natural medicines were developed to deal with illnesses. One of these herbal medicines was *Camelia Sinensis*, a tea. Now, drinking tea has become a habit worldwide, and its health benefits have been acknowledged. Through its anti-inflammatory, immunoregulatory, and antioxidant properties, tea protects against type 2 diabetes, obesity, cardiovascular conditions, cancer, and immune-related diseases (17–22). Epigallocatechin gallate (EGCG), a well-known catechin, is one of the most evaluated components of tea. EGCG inhibits the secretion of inflammatory cytokines, inhibits the main protease (MPro) of SARS-CoV-2 (23), and interferes with spike binding to ACE2 receptors (24, 25). Many *in silico* evaluations found that the oolongobobisflavan-A molecule had the potential to inhibit the bioactivity of Mpro of SARS-CoV-2 (26). Barrigenol, kaempferol, and myricetin had good docking scores in inhibiting non-structural protein 15 (Nsp15) of SARS-CoV-2 (27). Theaflavin could potentially inhibit non-structural protein 16 (Nsp16) of SARS-CoV-2 (28); epicatechin-3,5-di-O-gallate, epigallocatechin-3,5-di-O-gallate, and epigallocatechin-3,4-di-O-gallate had better inhibitory effects on the enzyme RNA-dependent RNA polymerase (RdRp) than some repurposed drug molecules (29).

An *in vitro* study found that green tea in general and EGCGs in particular significantly inhibit the entry to cells and post-entry stages of the viral life cycle while suppressing the activity of SARS-CoV-2 protease (30, 31). However, a majority of published *in vitro* experiments assessed the viricidal properties of some components of tea leaf extracts at very high, supraphysiological concentrations (32, 33). Therefore, whether tea drinking could impact the susceptibility or severity of COVID-19 in virus-exposed human populations is not known. Exploring the link between tea intake and COVID-19 may lead to an improvement in the management of coronavirus infection. The Mendelian randomization (MR) framework may be used to infer a potential causative association between a phenotype (exposure) that can be genetically influenced and a disease outcome. To achieve this, genetic variants are utilized as instrumental variables (34). The MR framework has been widely used in recent studies to explore relationships between related phenotypes (35–39). This study used the MR approach to test whether habitual tea intake exerts any causal influences on contracting SARS-CoV-2 and severe outcomes of COVID-19.

## Materials and methods

### Study design and data sources

The study was based on publicly available genome-wide association study (GWAS) summary results. The summary statistics for the outcomes of COVID-19 were obtained from the COVID-19. Host Genetics Initiative (HGI) GWAS meta-analysis round 7, including SARS-CoV-2 infection (122,616 cases and 2,475,240 controls), hospitalized COVID-19 (32,519 cases and 2,062,805 controls), and critical COVID-19 (13,769 cases and 1,072,442 controls) (40). The SARS-CoV-2 infection dataset mainly reflects the overall susceptibility to the virus, whereas the hospitalized and critical COVID-19 datasets represent the severity of the disease. Therefore, we collectively called the latter two outcomes “severe COVID-19.” The tea intake GWAS dataset included 441,279 participants from the UK Biobank (UKB) (41), which was obtained from YangLab (42). All participants in the GWAS datasets were from the European population. Ethical approvals were obtained from the original studies.

### MR analyses

The main analyses were performed by using the inverse-variance weighted (IVW) method, complemented with the weighted median and MR-Egger methods, and implemented in TwoSampleMR (43). The intercept from the MR-Egger regression was utilized to evaluate the average horizontal pleiotropy (44). The IVW model was used as the main statistical method.

### Sensitivity analyses

The heterogeneity in the MR analysis was evaluated by Cochran's *Q*-test and *I*^2^ statistics (both *P* < 0.05 and *I*^2^ > 0.25) (45). The significant associations between tea intake and COVID-19 were determined by IVW-based *P* < 0.017 (0.05/3). Single-nucleotide polymorphisms (SNPs) with genome-wide significance (*P* < 5 × 10^−8^) in the tea intake dataset were selected as instrumental variables (IVs) and further pruned using a clumping *r*^2^ cutoff of 0.01 within a 10 Mb window. For each MR analysis, we removed SNPs not present in the outcome dataset and palindromic SNPs with intermediate allele frequencies. We harmonized each pair of exposure and outcome datasets by aligning the effect allele for exposure and outcome with the obtained variant effects and standard errors of each dataset.

We conducted the MR analyses in R (version 4.0.5) (46).

## Results

### MR analysis

**Figure 1:**
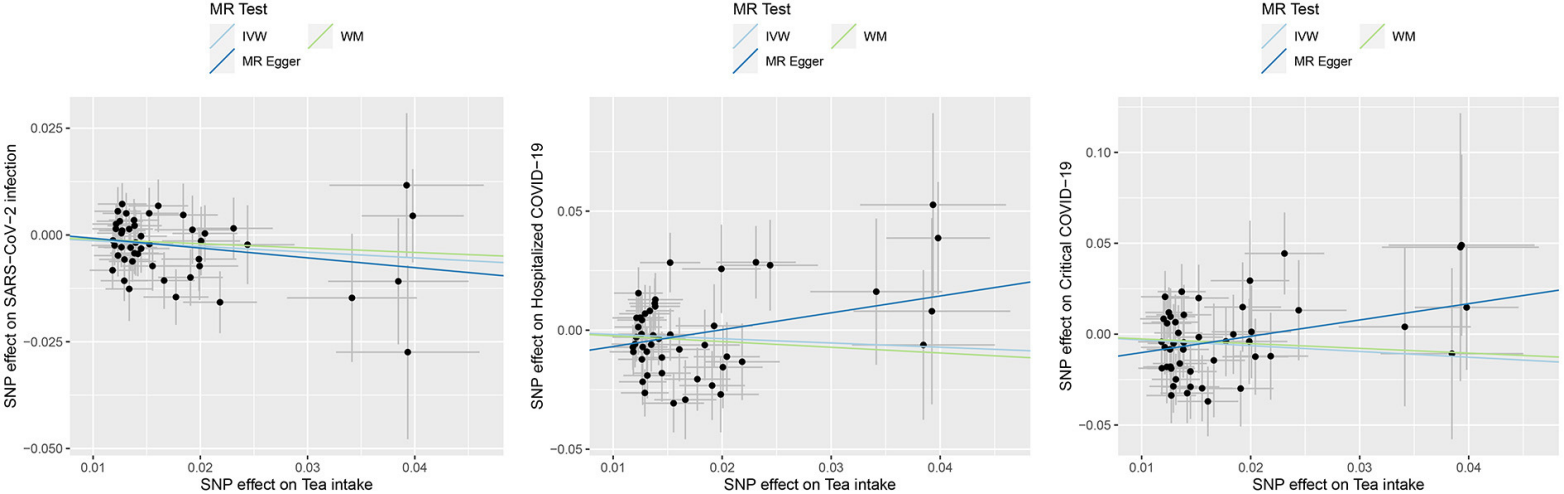
Causal effects of tea intake on the COVID-19 outcomes. Scatter plot for the SNP effect size estimate. Relationship between the SNP effect size estimate of tea intake (x-axis) and the corresponding effect size estimate of outcomes of COVID-19 (y-axis). The trait on the x-axis denotes exposure, the trait on the y-axis denotes outcome, and each cross point represents an instrumental variant. The dots and short lines through the dots denote the effect sizes (b) and the 95% CI of tea intake on the outcome of COVID-19. IVW, inverse variance weighted; WM, weighted median.

In the MR analysis of the causal effects of tea intake on the COVID-19 outcomes, a total of 47 IVs were derived. Our MR-Egger analysis showed that genetic propensity for tea intake conferred a causal protective effect on susceptibility to SARS-CoV-2 infection [OR: 0.87, 95% confidence interval (CI): 0.78–0.97, *P* = 0.015]. However, no causal effects of tea intake on hospitalized COVID-19 (0.84, 0.64–1.10, *P* = 0.201) or critical COVID-19 (0.73, 0.51–1.03, *P* = 0.074) were detected (Table 1, Figure 1).

**Table 1:** Causal effects of tea intake on the COVID-19 outcomes.

**Outcome**	**Method**	**b (se)**	**OR [95%CI]**	**N_IV**	**Q_P**	**I2**	**Egger_** **intercept**	**P_** **pleiotropy**	**P**
Critical COVID-19	IVW	−0.317 (0.178)	0.73 [0.51–1.03]	47	0.371	0.052	NA	NA	0.074
Critical COVID-19	WM	−0.261 (0.248)	0.77 [0.47–1.25]	47	NA	NA	NA	NA	0.294
Critical COVID-19	MR-Egger	0.897 (0.641)	2.45 [0.70–8.60]	47	0.486	−0.030	−0.019	0.055	0.168
Hospitalized COVID-19	IVW	−0.179 (0.140)	0.84 [0.64–1.10]	47	0.026	0.308	NA	NA	0.201
Hospitalized COVID-19	WM	−0.240 (0.170)	0.79 [0.56–1.10]	47	NA	NA	NA	NA	0.158
Hospitalized COVID-19	MR-Egger	0.708 (0.510)	2.03 [0.75–5.51]	47	0.047	0.258	−0.014	0.078	0.172
SARS-CoV-2 infection	IVW	−0.134 (0.055)	0.87 [0.78–0.97]	47	0.632	−0.090	NA	NA	0.015
SARS-CoV-2 infection	WM	−0.102 (0.080)	0.90 [0.77–1.06]	47	NA	NA	NA	NA	0.201
SARS-CoV-2 infection	MR-Egger	−0.229 (0.205)	0.80 [0.53–1.19]	47	0.600	−0.096	0.002	0.632	0.270

IVW, inverse variance weighted; WM, weighted median; OR, odds ratio; CI, confidence interval; N_IV, number of instrumental variables; Q_P, Cochran's P-value of heterogeneity analysis.

### Sensitivity analysis

For the causal association between tea intake and SARS-CoV-2 infection, the directions of causal effect estimates across the three methods were largely the same. The effect sizes between tea intake and SARS-CoV-2 were 0.87 (0.78–0.97) for the IVW model, 0.90 (0.77–1.06) for the WM model, and 0.80 (0.53–1.19) for the MR-Egger model (Table 1, Figure 1). There was no evidence supporting the heterogeneity concerning the effects of habitual tea intake on SARS-CoV-2 infection critical COVID-19 (Cochran's *P* = 0.632, *I*^2^ < 0.25; Table 1, Figure 1).

The effect sizes between tea intake and hospitalized COVID-19 were 0.84 (0.64–1.10) for the IVW model, 0.79 (0.56–1.10) for the WM model, and 2.03 (0.75–5.51) for the MR-Egger model (Table 1, Figure 1). There was evidence suggesting heterogeneity of the causal association between tea intake and hospitalized COVID-19 (Cochran's *P* = 0.026, *I*^2^ = 0.308).

The effect sizes between tea intake and critical COVID-19 were 0.73 (0.51–1.03) for the IVW model, 0.77 (0.47–1.25) for the WM model, and 2.45 (0.70–8.60) for the MR-Egger model (Table 1, Figure 1). The heterogeneity analysis did not support the heterogeneity of the causal association (Cochran's *P* = 0.371, *I*^2^ = 0.052).

Notably, tests of MR-Egger regression did not support the directional pleiotropy of the genetic IVs for the MR analyses (MR-Egger intercept < 0.02, *P* > 0.05).

## Discussion

Tea drinking is commonly regarded as a convenient and safe complementary therapy and healthy habit. Recent modeling studies have shown that various tea components are highly effective in blocking SARS-CoV-2 infection (24, 25, 47, 48). In addition, tea polyphenols might suppress the virus through their beneficial effects on intestinal microbiota (49). It has yet to be determined whether habitual tea consumption may exert an effect against COVID-19 in exposed populations.

Here, we provide convincing evidence for the potential influence of tea intake on the COVID-19 outcomes. In our study, a 1-SD increase in tea intake was associated with a 13% decreased risk for SARS-CoV-2 infection. The anti-COVID-19 effect of tea intake might be due to its inhibition of viral binding and/or enhancement of the human innate immune response (50). Green or black tea infusions are rich in bioactive nutrients: polyphenols, EGCG, theanine, theaflavin, alkaloids, caffeine, and its intermediates, theophylline, theobromine, and others, many of which have immunomodulatory or anti-inflammatory properties (51). Notably, the consumption of tea is safe for humans even at high levels, with adverse effects being rare (52). Our findings conclusively support the notion of possible protective effects of tea consumption against symptomatic COVID-19 (53, 54).

However, as our findings showed, routine tea intake was not associated with a decrease in the risks of COVID-19 hospitalization or critical COVID-19. These two severe outcomes are commonly due to a strong immunological response to the virus. It is necessary to explore whether the therapeutic effects of tea are related to its particular phytochemicals or a general habit of high tea intake. The full realization of the therapeutic potential of *Camellia sinensis* may require the development of advanced delivery systems for its molecular constituents.

Individual phytochemicals of *C. sinensis* should be studied as possible dietary supplements suitable for the time of pandemics. As the effectiveness of EGCG and other polyphenols in tea is limited due to their low oral bioavailability, further studies on the advanced delivery of individual tea constituents are warranted.

The main strength of the study was that MR analysis is less affected by the causality pitfalls, namely, the presence of confounding factors and reverse causation, which is commonly seen in traditionally designed observational studies. Here, the largest available GWAS summary datasets were utilized for tracing the causative association between COVID-19 and tea intake. Our study had several limitations. In particular, we assessed only genetic effects with no regard to the effects of the environment, which are critical for both tea intake and COVID-19. MR analyses might be biased due to pleiotropy, especially in non-homogenous datasets. To mitigate the latter, we tested the MR assumptions using various models.

## Conclusion

In summary, our MR-based study revealed a causal protective effect of habitual tea intake on symptomatic SARS-CoV-2 infections but no protective effect against severe COVID-19. Our study clarifies that the protection provided by tea intake is related to building the resilience of the human body rather than to direct therapeutic correction of entrenched pathology. The uncovered protective effects should be dissected further to determine the influences of particular tea leaf constituents, including EGCG, and the behavioral or general health-related traits associated with tea consumption. Encouraging tea consumption might be a cost-effective natural measure for the prevention of COVID-19 transmission in humans.

## Data availability statement

The raw data supporting the conclusions of this article will be made available by the authors, without undue reservation.

## Author contributions

FZ conceived the project, supervised the study, and analyzed the data. FZ, WY, HC, YS, and AB wrote the manuscript. All authors read and approved the final manuscript.
